# Postoperative seizures in meningioma patients: improving patient selection for antiepileptic drug therapy

**DOI:** 10.1007/s11060-018-2941-2

**Published:** 2018-06-29

**Authors:** Abdurrahman I. Islim, Arousa Ali, Ananyo Bagchi, Mohammad U. Ahmad, Samantha J. Mills, Emmanuel Chavredakis, Andrew R. Brodbelt, Michael D. Jenkinson

**Affiliations:** 1 Institute of Translational Medicine, University of Liverpool, Liverpool, UK; 2 School of Medicine, University of Liverpool, Liverpool, UK; 3 Department of Neurosurgery, The Walton Centre NHS Foundation Trust, Liverpool, UK; 4 Department of Neuroradiology, The Walton Centre NHS Foundation Trust, Liverpool, UK; 5 Aintree University Hospital NHS Foundation Trust, Liverpool, UK

**Keywords:** Antiepileptic drugs, Epilepsy, Meningioma, Post-operative seizure

## Abstract

**Background:**

Epilepsy is a major cause of morbidity and mortality in meningioma patients. The aims of this study were to determine which factors predispose meningioma patients to developing perioperative seizures and to understand the impact of antiepileptic drugs.

**Methods:**

Patients treated for a histologically-confirmed intracranial meningioma at the authors’ institution between 2010 and 2015 were retrospectively examined. Clinical and imaging data were assessed. Multivariate analysis was performed using binary logistic regression. The effect of antiepileptic treatment was assessed using survival analysis.

**Results:**

Two hundred and eighty-three patients met the selection criteria; seizures were present in 68 preoperatively (24%) and in 48 patients (17%) following surgery. Of the 68 with preoperative seizures, 19 continued to have them, whereas de-novo seizures arose postoperatively in 29 seizure-naïve patients. Risk factors of postoperative seizures were convexity location (OR 2.05 [95% CI 1.07–3.98], p = 0.030), fronto-parietal location (OR 4.42 [95% CI 1.49–13.16], p = 0.007) and preoperative seizures (OR 2.65 [95% CI 1.37–5.24], p = 0.005). The two locations, in addition to the presence of midline shift on preoperative imaging (OR 4.15 [95% CI 1.54–11.24], p = 0.005), were significantly correlated with postoperative seizures in seizure-naïve patients. Antiepileptic treatment in patients with those risk factors reduced the possibility of seizures at any time point within the 1st year postoperatively by approximately 40%, although this did not meet statistical significance.

**Conclusion:**

Prophylactic antiepileptic treatment might be warranted in seizure-naïve meningioma patients with ≥ 1 risk factor. High-quality randomised controlled trials are required to verify those factors and to define the role of antiepileptics in meningioma practice.

## Introduction

Whilst focal neurological deficits and incidental discovery account for the majority of new diagnoses of intracranial meningioma [1, 2], approximately a third of patients present with focal epilepsy [3]. Antiepileptic drugs (AEDs) are indicated for the treatment of brain tumour-related seizures, however, there still remains no consensus on whether prophylactic AEDs should be prescribed in seizure-naïve patients to prevent the development of postoperative seizures [4]. Epilepsy in meningioma patients is a major cause of morbidity and mortality [5, 6], but the rate at which new seizures develop in patients undergoing meningioma surgery varies, and the efficacy of AEDs in reducing post-operative seizure rates remains questionable [7, 8]. Furthermore, drug-related side effects, which can impair quality of life (QoL) and neurocognitive function (NCF), occur in up to half of patients [5, 9, 10]. Therefore, appropriate selection of patients at risk of developing epilepsy in the peri-operative period and who might benefit from AED treatment for meningioma resection is important.

## Objectives

To investigate the risk factors associated with developing peri-operative epilepsy in meningioma patients, and to determine whether AEDs reduce the risk of postoperative seizures.

## Methods

### Patient selection

Data for patients who underwent craniotomy and resection of a histologically-confirmed intracranial meningioma between January 2010 and December 2015 were collected retrospectively. Eligibility criteria were as follows: (i) surgery for newly-diagnosed meningioma, (ii) a follow-up period ≥ 12 months, (iii) pre- and postoperative imaging available.

### Clinical and radiological characteristics

Clinical information was obtained from the medial records. Extracted preoperative data included patient demographics, seizure status and semiology (categorised according to the International League Against Epilepsy [ILAE] 2017 classification [11]), the use of AEDs (treatment or prophylaxis), clinical presentation (headache and focal neurological deficits), and the Eastern Cooperative Oncology Group performance status (ECOG). Radiological factors, recorded using the Carestream Vue picture archiving and communication system (PACS) version 11, included tumour location and volume, peritumoural signal change, midline shift, and calcification. Tumour volume was determined by manual segmentation on gadolinium-enhanced T1-weighted MRI scans. Peritumoural signal change was assessed in relation to tumour volume on T2-weighted MRI and grouped as follows: 0–5, 6–33, 34–66 and 67–100%. Zero to 5% was defined as absent oedema (based on the Visually AcceSAble Rembrandt Images [VASARI] MR features for gliomas [12]).

Tumours were classified according to the WHO 2007 system. Extent of resection (as recorded by the neurosurgeon in the operative notes), the presence of residual tumour on contrast-enhanced postoperative MRI, post-craniotomy complications (hydrocephalus, CNS infection, clinically symptomatic haemorrhage and radiological haemorrhage), and the incidence of postoperative seizures were recorded. Gross total resection (GTR) was defined as Simpson grades I–III, whilst subtotal resection (STR) was defined as grades IV–V. For patients who had postoperative seizures, time to first seizure was calculated from the date of surgery to the first clinical encounter where seizure signs and symptoms were reported and judged by the attending neurosurgeon/neurologist to have constituted an epileptic seizure. Complete seizure control, which equates to a postoperative ILAE outcome of 1 [13], was determined at 12 months following this encounter.

### Data analysis

Data was analysed using SPSS v24.0 (IBM, Armonk, NY, USA).

Patients were stratified based on the presence of preoperative seizures. Clinical correlates of preoperative epilepsy were accordingly determined using binary logistic regression (BLR), incorporating only factors with a significance level < 0.05 on univariate analysis, which was performed using Pearson’s Chi square test for categorical variables and the Mann–Whitney U test for continuous variables. Odds ratios (ORs) with 95% confidence intervals (CI) were calculated to assess factors’ effect size. Risk factors of postoperative seizures were similarly determined in all patients, and in seizure-naïve patients.

A forward stepwise selection procedure was utilised to determine the model of best fit. Model assumptions were tested by examination of residuals and the overall fit was assessed using the Hosmer and Lemeshow (H–L) test and the area under the receiver operating characteristic (ROC) curve (AUC).

The effect of AED therapy on postoperative seizure rates was assessed using a cox proportional hazards regression model. As the Driver and Vehicle Licensing agency (DVLA) in the United Kingdom (UK) sets the driving ban to a maximum duration of 12 months postoperatively in meningioma patients, time to first seizure was censored at 365 days in case of no-occurrence within the first 12 months [14]. The model encompassed two factors: AED treatment (yes/no), and a dummy variable incorporating statistically significant variables in the corresponding BLR model. Model performance was assessed using the likelihood-ratio statistic (–2LL) and for each variable a hazard ratio (HR) was extracted with its 95% CI.

### Data validation

For tumour volume, Bland–Altman plots were performed to assess inter- and intra-observer variability. The repeated measurements were carried out on a random sample of 11 patients.

Bivariate correlation was undertaken to assess the relationships between different factors. Baseline variables that proved to be significantly correlated (p < 0.05) were entered as one into the BLR model.

Distribution of continuous variables was examined with normally distributed variables expressed as mean (standard deviation [SD]) and skewed variables as median (interquartile range [IQR]). Statistically significant skewed variables (p < 0.05) were transformed into their natural logs before being inputted into the BLR model.

### Meningioma surgery and AED practice

No protocol for AED treatment is available at the authors’ institution and practices are based on surgeon preference. Management decisions for meningioma are by consensus within the neuro-oncology multidisciplinary team. Patients are considered for surgery if symptomatic or asymptomatic and showing evidence of meningioma growth on surveillance imaging. Age, performance status and comorbidities are also considered. Of note, none of the patients included in this study were subject to preoperative embolisation.

## Results

### Study population

**Fig. 1:**
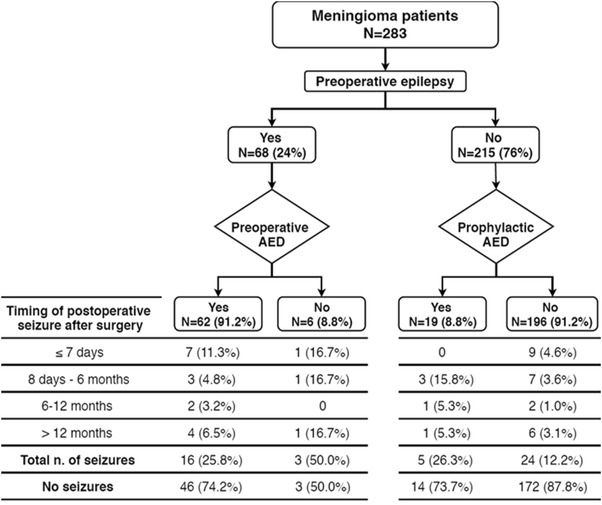
The study flow chart

Demographic and clinical data are summarised in Table 1. Two hundred and eighty-three patients met the inclusion criteria. Sixty-eight patients presented with seizures, 62 of whom received preoperative AED treatment. The remaining 215 patients were seizure-naïve at presentation and 19 received prophylactic AED treatment (Fig. 1). Postoperative seizures were observed in 17% (48/283). Median time to seizure occurrence was 58 days (IQR = 442). There was one postoperative death due to epilepsy in a 69-year old male (ECOG 0, presented with epilepsy and treated with lamotrigine). Following surgical resection of a frontal convexity meningioma, the patient had a focal to bilateral seizure on day 5 after surgery and subsequently died. No other seizure-related mortalities occurred.

**Table 1:** Demographic and clinical data for meningioma patients and univariate analysis of preoperative seizures

	Total no. of patients	Preoperative seizures		OR (95% CI)	P
	(N = 283)	Yes (N = 68)	No (N = 215)		
Focal-aware					
Motor (%)		16 (23.5)			
Non-motor (%)		9 (13.2)			
Focal-impaired awareness (%)		7 (10.3)			
Focal to bilateral (%)		36 (52.9)			
Age at diagnosis (years)					
Mean (SD)	57.7 (13.0)	56.2 (14.1)	58.2 (12.6)		0.410
Gender (%)					
Female	214 (75.6)	47 (69.1)	167 (77.7)		
Male	69 (24.4)	21 (30.9)	48 (22.3)	1.56 (0.85–2.85)	0.152
WHO grade (%)					
I	233 (82.3)	54 (79.4)	179 (83.3)		
II	47 (16.6)	13 (19.1)	34 (15.8)	1.29 (0.65–2.57)	0.469
III	3 (1.1)	1 (1.5)	2 (0.9)	1.59 (0.14–17.81)	0.707
Tumour location (%)					
Non-skull base	207 (73.1)	49 (72.1)	158 (73.5)	0.93 (0.51–1.71)	0.871
Convexity	98 (34.6)	25 (36.8)	73 (34.0)	1.13 (0.64–1.20)	0.671
Parafalcine	39 (13.8)	15 (22.1)	24 (11.2)	2.25 (1.10–4.60)	0.023
Tentorial	24 (8.5)	2 (2.9)	22 (10.2)	0.27 (0.06–1.16)	0.060
Convexity/parafalcine	17 (6.0)	2 (2.9)	15 (7.0)	0.40 (0.09–1.81)	0.222
Parasagittal	12 (4.2)	4 (5.9)	8 (3.7)	1.61 (0.47–5.55)	0.441
Posterior fossa	5 (1.8)	0	5 (2.3)	N/A	N/A
Others	12	1	11	N/A	N/A
Skull base	76 (26.9)	19 (27.9)	57 (26.5)		
Sphenoid	34 (12.0)	10 (14.7)	24 (11.2)	1.37 (0.62–3.04)	0.433
Olfactory groove	18 (6.4)	5 (7.4)	13 (6.0)	1.23 (0.42–3.40)	0.700
Suprasellar	10 (3.5)	1 (1.5)	9 (4.2)	0.34 (0.04–2.75)	0.290
Posterior fossa	2 (0.7)	0	2 (0.9)	N/A	N/A
Others	12	3	9	N/A	N/A
Side					
Right	123 (43.5)	36 (52.9)	87 (40.5)	1.66 (0.96–2.86)	0.070
Left	125 (44.2)	27 (39.7)	98 (45.6)	0.79 (0.45–1.37)	0.395
Bilateral	35 (12.4)	5 (7.4)	30 (14.0)	0.49 (0.18–1.32)	0.150
Relation to brain lobes					
Frontal	138 (48.8)	37 (54.4)	101 (47.0)	1.35 (0.78–2.33)	0.285
Parietal	34 (12.0)	17 (25.0)	17 (7.9)	3.88 (1.85–8.13)	< 0.001
Temporal	29 (10.3)	8 (11.8)	21 (9.8)	1.23 (0.52–2.92)	0.636
Fronto-parietal	16 (5.7)	3 (4.4)	13 (6.0)	0.72 (0.19–2.59)	0.611
Fronto-temporal	12 (4.2)	0	12 (5.6)	N/A	N/A
Occipital	9 (3.2)	1 (1.5)	8 (3.7)	0.39 (0.05–3.14)	0.357
Preoperative radiological characteristics					
Tumour volume (cm^3^)^b^ (%)					
Median (IQR)	24.8 (37.8)	28.1 (27.7)	37.2 (39.8)		0.195
≤ 10 cm^3^	57 (20.5)	12 (17.9)	45 (21.3)		
> 10 cm^3^	221 (79.5)	55 (82.1)	166 (78.7)	1.24 (0.61–2.52)	0.546
Midline shift (%)					
Yes	155 (54.8)	40 (58.8)	115 (53.5)	1.24 (0.72–2.16)	0.441
No	128 (45.2)	28 (41.2)	100 (46.5)		
Calcification^a^ (%)					
Yes	66 (23.3)	15 (22.1)	51 (23.7)		
No	216 (76.3)	53 (77.9)	163 (75.8)	1.11 (0.57–2.13)	0.815
Presence of peritumoural signal intensity (%)					
6–100% (present)	158 (55.8)	54 (79.4)	104 (48.4)	4.12 (2.16–7.85)	< 0.001
0–5% (absent)	125 (44.2)	14 (20.6)	111 (51.6)		
Preoperative headaches (%)					
Yes	125 (44.2)	26 (38.2)	99 (46.0)		
No	158 (55.8)	42 (61.8)	116 (54.0)	1.38 (0.79–2.41)	0.258
Preoperative neurological deficits (%)					
Yes	201 (71.0)	33 (48.5)	168 (78.1)		
No	82 (29.0)	35 (51.5)	47 (21.9)	3.79 (2.13–6.74)	< 0.001
Preoperative ECOG performance status (%)					
0–2	264 (93.3)	63 (92.6)	201 (93.5)		
3–4	19 (6.7)	5 (7.4)	14 (6.5)	0.71 (0.34–1.51)	0.370

*WHO* World Health Organisation, *ECOG* Eastern Cooperative Oncology Group

^a^Missing 1 value

^b^Missing 5 values

### Predictors of preoperative seizures

Univariate analysis (Table 1) revealed parafalcine and parietal tumour locations to be associated with preoperative seizures (p = 0.023, p < 0.001), however, the two factors were linked on bivariate correlation (p = 0.022) and were therefore incorporated as one variable into the BLR model. The presence of peritumoural signal intensity (6–100%) and the absence of focal neurological deficits were also correlated with preoperative seizures (p < 0.001, p < 0.001).

All three factors remained significant in the BLR model: parietal–parafalcine location (OR 2.81 [95% CI 1.44–5.46], p = 0.002), peritumoural signal change (OR 5.10 [95% CI 2.49–10.52], p < 0.001) and the absence of focal neurological deficits (OR 5.55 [95% CI 2.63–11.11], p < 0.001).

### Predictors of postoperative seizures

#### Whole study population

On univariate analysis, convexity location (p = 0.014), fronto-parietal location (p = 0.003), preoperative seizures (p = 0.006) and the presence of peritumoural signal intensity (6–100%) (p = 0.022) were significantly associated with postoperative seizures (Table 2). The two meningioma locations were not correlated (p = 0.19). All four factors were inserted into the BLR model, in which the following remained significant: convexity location (OR 2.05 [95% CI 1.07–3.98], p = 0.030), fronto-parietal location (OR 4.42 [95% CI 1.49–13.16], p = 0.007) and preoperative seizures (OR 2.65 [95% CI 1.37–5.24], p = 0.005).

**Table 2:** Analysis of risk factors for postoperative seizures

Characteristic	All patients (N = 283)				Seizure-naïve patients (N = 215)			
	Postoperative seizures		Univariate analysis		Postoperative seizures		Univariate analysis	
	Yes (N = 48)	No (N = 235)	OR (95% CI)	P	Yes (N = 29)	No (N = 186)	OR (95% CI)	P
Age at diagnosis (years)								
Mean (SD)	57.6 (11.4)	57.7 (13.3)		0.751	59.0 (11.9)	58.1 (12.7)		0.744
Gender (%)								
Female	31 (64.6)	183 (77.9)			17 (58.6)	150 (80.6)		
Male	17 (35.4)	52 (22.1)	1.93 (0.99–3.76)	0.051	12 (41.4)	36 (19.4)	2.94 (1.29–6.70)	0.008
WHO grade (%)								
I	39 (81.3)	194 (82.6)			23 (79.3)	156 (83.9)		
II	8 (16.7)	39 (16.6)	1.09 (0.49–2.43)	0.829	6 (20.7)	28 (15.1)	1.36 (0.51–3.61)	0.541
III	1 (2.1)	2 (0.9)	2.48 (0.22–27.89)	0.462	0	2 (1.1)	N/A	N/A
Tumour location (%)								
Non-skull base	37 (77.1)	170 (72.3)	1.29 (0.62–2.67)	0.499	24 (82.8)	134 (72.0)	1.86 (0.67–5.14)	0.224
Convexity	24 (50.0)	74 (31.5)	2.18 (1.16–4.08)	0.014	17 (58.6)	56 (30.1)	2.83 (1.27–6.34)	0.003
Parafalcine	10 (20.8)	29 (12.3)	1.87 (0.84–4.15)	0.120	6 (20.7)	18 (9.7)	2.43 (0.87–6.76)	0.080
Tentorial	1 (2.1)	23 (9.8)	0.20 (0.03–1.49)	0.115	0	22 (11.8)	N/A	N/A
Convexity/parafalcine	1 (2.1)	16 (6.8)	0.29 (0.04–2.25)	0.209	0	15 (8.1)	N/A	N/A
Parasagittal	1 (2.1)	11 (4.7)	0.43 (0.05–3.44)	0.416	1 (3.4)	7 (3.8)	0.91 (0.11–7.71)	0.934
Posterior fossa	0	5 (2.1)	N/A	N/A	0 (0.0)	5 (2.7)	N/A	N/A
Others	0	12	N/A	N/A	0	11	N/A	N/A
Skull base	11 (22.9)	65 (27.7)			5 (17.2)	52 (28.0)		
Sphenoid	7 (14.6)	27 (11.5)	1.32 (0.54–3.22)	0.548	4 (13.8)	20 (10.8)	1.33 (0.42–4.21)	0.629
Olfactory groove	1 (2.1)	17 (6.0)	0.27 (0.03–2.10)	0.183	0	13 (7.0)	N/A	N/A
Suprasellar	0	10 (4.3)	N/A	N/A	0	9 (4.8)	N/A	N/A
Posterior fossa	0	2 (0.9)	N/A	N/A	0	2 (1.1)	N/A	N/A
Others	3	9	N/A	N/A	1	8	N/A	N/A
Side								
Right	20 (41.7)	103 (43.8)	0.92 (0.49–1.72)	0.783	10 (34.5)	77 (41.4)	0.75 (0.33–1.69)	0.480
Left	26 (54.2)	99 (42.1)	1.62 (0.87–3.03)	0.126	18 (62.1)	80 (43.0)	2.17 (0.97–4.85)	0.059
Bilateral	2 (4.2)	33 (14.0)	0.27 (0.06–1.15)	0.058	1 (3.4)	29 (15.6)	0.19 (0.03–1.48)	0.113
Relation to brain lobes								
Frontal	25 (52.1)	113 (48.1)	1.18 (0.63–2.18)	0.614	16 (55.2)	85 (36.2)	1.46 (0.67–3.21)	0.342
Parietal	5 (10.4)	29 (12.3)	0.83 (0.30–2.26)	0.709	1 (3.4)	16 (8.6)	0.38 (0.05–2.98)	0.339
Temporal	7 (14.6)	22 (9.4)	1.65 (0.66–4.12)	0.277	4 (13.8)	17 (9.1)	1.59 (0.50–5.11)	0.432
Fronto-parietal	7 (14.6)	9 (3.9)	4.35 (1.52–12.50)	0.003	6 (20.7)	7 (3.7)	6.67 (2.06–21.57)	< 0.001
Fronto-temporal	1 (2.1)	11 (4.7)	0.43 (0.05–3.44)	0.416	1 (3.4)	11 (5.9)	0.57 (0.07–4.57)	0.591
Occipital	0	9 (3.3)	N/A	N/A	0	8 (4.3)	N/A	N/A
Preoperative radiological characteristics								
Tumour volume (cm^3^)^c^ (%)								
Median (IQR)	47.5 (56.3)	32.5 (35.4)		0.060	63.3 (70.3)	33.2 (36.6)		0.003
≤ 10 cm^3^	6 (12.8)	51 (22.6)			3 (10.7)	42 (23.0)		
> 10 cm^3^	41 (87.2)	180 (77.9)	1.94 (0.78–4.82)	0.149	25 (89.3)	141 (77.0)	2.48 (0.71–8.63)	0.141
Midline shift (%)								
Yes	30 (62.5)	125 (53.2)	1.47 (0.78–2.77)	0.238	21 (72.4)	94 (50.5)	2.56 (1.09–6.25)	0.028
No	18 (37.5)	110 (46.8)			8 (27.6)	92 (49.5)		
Calcification^a^ (%)								
Yes	13 (27.1)	53 (22.6)			9 (31.0)	42 (22.6)		
No	34 (70.8)	182 (77.4)	0.76 (0.38–1.55)	0.450	19 (65.5)	144 (77.4)	0.62 (0.26–1.46)	0.268
Presence of peritumoural signal intensity (%)								
6–100% (present)	34 (70.8)	124 (52.8)	2.17 (1.11–4.35)	0.022	20 (69.0)	84 (45.2)	2.70 (1.16–6.25)	0.017
0–5% (absent)	14 (29.2)	111 (47.2)			9 (31.0)	102 (54.8)		
Preoperative headaches (%)								
Yes	17 (35.4)	108 (46.0)			12 (41.4)	87 (46.8)		
No	31 (64.6)	127 (54.0)	1.55 (0.81–2.95)	0.180	17 (58.6)	99 (53.2)	1.24 (0.56–2.75)	0.588
Preoperative neurological deficits (%)								
Yes	30 (62.5)	175 (74.5)			24 (82.8)	144 (77.4)		
No	18 (37.5)	60 (25.5)	1.75 (0.91–3.37)	0.093	5 (17.2)	42 (22.6)	0.71 (0.26–1.99)	0.518
Preoperative ECOG performance status (%)								
0–2	44 (91.7)	220 (93.6)			26 (89.7)	175 (94.1)		
3–4	4 (8.3)	15 (6.4)	1.41 (0.66–2.99)	0.373	3 (10.3)	11 (5.9)	2.09 (0.87–4.99)	0.093
Simpson grade of resection (%)								
GTR	42 (87.5)	191 (81.3)	1.61 (0.65–4.03)	0.303	26 (89.7)	150 (80.6)	2.08 (0.59–7.25)	0.242
1	16 (33.3)	49 (20.9)	1.95 (0.98–3.84)	0.054	11 (37.9)	36 (19.4)	2.63 (1.14–5.88)	0.020
2	24 (50.0)	129 (54.9)	0.82 (0.44–1.53)	0.535	15 (51.7)	103 (55.4)	0.86 (0.39–1.89)	0.713
3	2 (4.2)	13 (5.5)	0.75 (0.16–3.40)	0.701	0	11 (5.9)	N/A	N/A
STR	6 (12.5)	44 (18.7)			3 (10.3)	36 (19.4)		
4	6 (12.5)	44 (18.7)			3 (10.3)	36 (19.4)		
5	0	0	N/A	N/A	0	0	N/A	N/A
Postoperative residual tumour^b^ (%)								
Yes	7 (14.9)	43 (18.5)			3 (10.7)	32 (17.3)		
No	40 (85.1)	189 (81.5)	1.30 (0.55–3.10)	0.554	25 (89.3)	153 (82.7)	1.74 (0.50–6.12)	0.381
Postoperative neurosurgical complications^b^ (%)								
Yes	28 (58.3)	110 (46.8)	1.63 (0.86–3.09)	0.128	19 (65.5)	91 (48.9)	1.94 (0.86–4.40)	0.108
No	19 (39.6)	122 (51.9)			10 (34.5)	93 (50.0)		
Radiological haemorrhage^b^ (%)								
Yes	27 (56.3)	104 (44.3)	1.66 (0.88–3.13)	0.114	18 (62.1)	85 (45.7)	1.91 (0.85–4.26)	0.112
No	20 (41.7)	128 (54.5)			11 (37.9)	99 (53.2)		
Clinical haemorrhage (%)								
Yes	4 (8.3)	11 (4.7)	1.85 (0.56–6.08)	0.303	4 (13.8)	8 (4.3)	3.57 (1.00-12.50)	0.038
No	44 (91.7)	224 (95.3)			25 (86.2)	178 (95.7)		
CNS infection (%)								
Yes	4 (8.3)	9 (3.8)	2.28 (0.67–7.74)	0.174	4 (13.8)	9 (4.8)	3.15 (0.90-10.98)	0.072
No	44 (91.7)	226 (96.2)			25 (86.2)	177 (95.2)		
Hydrocephalus (%)								
Yes	1 (2.1)	5 (2.1)	0.98 (0.11–8.57)	0.985	1 (3.4)	5 (2.7)	1.29 (0.15–11.48)	0.817
No	47 (97.9)	230 (97.9)			28 (96.6)	181 (97.3)		
Preoperative seizures (%)								
Yes	19 (39.6)	49 (20.9)	2.50 (1.28–4.76)	0.006	0	0	N/A	N/A
No	29 (60.4)	186 (79.1)			29 (100)	186 (100)		

*WHO* World Health Organisation, *ECOG* Eastern Cooperative Oncology Group, *GTR* gross total resection, *STR* subtotal resection, *CNS* central nervous system

^a^Missing 1 value

^b^Missing 4 values

^c^Missing 5 values

#### Seizure-naïve patients

Convexity location (p = 0.003), fronto-parietal location (p < 0.001), male sex (p = 0.008), midline shift (p = 0.028), presence of peritumoural signal intensity (6–100%) (p = 0.017), Simpson grade I resection (p = 0.020), and clinically symptomatic haemorrhage (p = 0.038) were statistically associated with postoperative seizures on univariate analysis (Table 2). The median meningioma volume in postoperative-seizure patients was 63.3 cm^3^ as opposed to 33.2 cm^3^ in patients who remained seizure-free (p = 0.003). Bland–Altman plots for intra-and inter-observer variability of meningioma volume indicated a good level of agreement.

Three factors remained significant in the BLR model: convexity location (OR 4.63 [95% CI 1.89–11.36], p < 0.001), fronto-parietal location (OR 7.52 [95% CI 2.04–27.78], p = 0.002), and the presence of midline shift on preoperative imaging (OR 4.15 [95% CI 1.54–11.24], p = 0.005).

### Models performance

H–L tests for the previous three models were > 0.05 indicating a good fit (0.27–0.83). AUC values and plotted residuals were acceptable for the 1st and 2nd model. Parameters of the 3rd model were poor.

### Antiepileptic drug treatment

The study flow chart (Fig. 1) outlines AED treatment arms and consequent seizure rates. The most frequently utilised AEDs were phenytoin (48.1%) and levetiracetam (25.9%). Prophylactic AED use in seizure-naïve patients who did not develop postoperative epilepsy ranged from a single dose at surgery to 1092 days (median = 275 [IQR = 419]). AEDs in patients with complete postoperative control of preoperative epilepsy, were stopped less than 12 months after surgery in 32 (65.3%) patients, whereas 17 (34.7%) were on lifelong treatment (> 12 months) (median = 351 [IQR = 1217]) (p = 0.185).

To examine the seizure response to AEDs, two cox regression analyses were performed: the first encompassing the whole study population and incorporating two variables: AED treatment and a dummy variable (convexity × fronto-parietal × preoperative seizures). The 2nd model comprised seizure-naïve patients and two variables were entered: AED treatment and one dummy variable (convexity × fronto-parietal × midline shift). The two models performed well (–2LLs = 0.001 and 0.004). Both dummy variables had HRs > 1 (p = 0.004, p = 0.002) whereas AED treatment in both models had a HR < 1, reducing adjusted seizure risk (≥ 1 risk factor), at any time within the 1st year postoperatively, by 38 and 37% respectively, albeit this did not reach statistical significance (p = 0.187, p = 0.451; Table 3).

**Table 3:** Cox regression model results

Model	Factor	HR (95% CI)	P
Whole study population	Preoperative AED	0.62 (0.31–1.26)	0.187
	Convexity × FP × preoperative seizures	1.06 (1.02–1.10)	0.004
Seizure-naïve patients	Preoperative AED	0.63 (0.20–2.05)	0.451
	Convexity × FP × midline shift	1.04 (1.01–1.06)	0.002

*FP* fronto-parietal location

### Twelve-month seizure control rates

One hundred and seventy-eight (90.8%) seizure-naïve patients who did not receive prophylactic AEDs remained seizure-free 12 months after surgery. The rate was slightly lower for seizure-naïve patients who were prescribed AEDs (78.9%) (p = 0.096). Fifty (80.6%) patients who had AED-treated preoperative epilepsy were free of seizures at 12 months as opposed to 4 (66.7%) untreated patients (p = 0.427). In total, the probability of seizure-freedom through 12 months of follow-up was 89.8% in seizure-naïve patients and 79.4% in patients with preoperative epilepsy (p = 0.029). These rates dropped to 86.5 and 72.1% respectively beyond 12 months (Fig. 1).

### Control of postoperative seizures within 12 months of their onset

Data was available in 47 patients (1 dead) and 18 (38.3%) had poorly controlled seizures. Ten out of 18 (55.6%) patients with poorly controlled epilepsy had seizures preoperatively. Of the 29 patients with controlled seizures, 8 (27.6%) patients had preoperative seizures (p = 0.015). At this stage, AED monotherapy was being used in 11/18 (61.1%) patients with poorly controlled seizures.

## Discussion

Studies addressing perioperative seizures are important for informing driving guidance and QoL in operated meningioma, and to justify the use or avoidance of prophylactic AEDs. In this cohort of 283 patients, parietal–parafalcine location, peritumoural signal change and the absence of focal neurological deficits were identified as independent predictors of preoperative seizures. Convexity and fronto-parietal locations, and the presence of preoperative seizures were significantly associated with postoperative seizures, in addition to the presence of midline shift on preoperative imaging in seizure-naïve patients. The likelihood of seizure-freedom after 12 months of follow-up was 89.7% in seizure-naïve patients and 79.4% in patients with preoperative epilepsy.

### Risk factors of preoperative seizures

In our study cohort, 24% of patients presented with seizures, which is higher than those rates of previous reports which comprised fewer non-skull base meningiomas [15, 16], and more specifically those located along the falx abutting the parietal lobe, a factor which retained significance in the BLR model pertaining to preoperative seizures.

The presence of peritumoural signal change, indicative of vasogenic oedema, and the absence of focal neurological deficits preoperatively were also independently associated with preoperative seizures, consistent with the findings of prior papers [16–19]. Oedema in meningioma patients is postulated to be the product of vascular endothelial growth factor-A and is more frequently observed in invasive subtypes of meningioma, although this did not prove to be the case in our study (WHO grade I: 23.2% vs. WHO grades II/III: 28%) [20, 21]. Smaller meningiomas, although statistically insignificant, were preoperatively more epileptogenic, potentially causing the development of seizures before symptoms of mass effect, such as focal neurological deficits, manifest. We postulate that smaller slow-growing meningiomas are allowed more time to disrupt the peritumoural functional environment driving epileptogenesis, whereas bigger relatively faster-growing meningiomas tend to display symptoms of mass effect before the epileptic process occurs.

### Predictive factors of postoperative seizures

De-novo seizures occurred in 29 seizure-naïve patients (13.5%), 9 (4.2%) of which arose in the early postoperative period (within 1 week of surgery), which is slightly higher than the pooled frequency of 2.7% in a recent systematic review [7]. Midline shift, previously shown to play a role in epilepsy development following evacuation of intracranial haemorrhages and resection of cerebral metastases [22, 23], was likewise independently associated with postoperative seizures in seizure-naïve meningioma patients.

In keeping with previous studies [18, 24, 25], tumour location was an independent predictive factor. Convexity and fronto-parietal locations increased the risk of seizures arising by two- and fivefold respectively, and these numbers were approximately doubled for seizure-naïve patients. The reason being the proximity to cortical areas which are susceptible to epilepsy-predisposing morphological and functional alterations [26]. This also holds for fronto-parietal meningiomas located in the vicinity of the hyperexcitable primary motor and somatosensory cortices, which had an associated epilepsy incidence rate of 63% in a previous study [27, 28].

Simpson grade I resection was correlated with postoperative seizures on univariate analysis. Most patients with Simpson I resection were convexity meningiomas in our cohort (bivariate correlation, p < 0.001), and these are considered more susceptible to postoperative seizures, therefore the association between Simpson resection and seizures is a statistical finding that is not clinically relevant.

The association between peritumoural oedema and postoperative seizures was noted on univariate analysis, however, it did not emerge as in independent factor in the BLR model. Due to the small number of patients with seizures (n = 48), we did not stratify into early and late epilepsy. Vasogenic oedema tends to resolve within 2 weeks of surgery in 90% of the cases and future studies should stratify patients into early and late seizures [29].

### Do AEDs have a role in reducing seizure rates postoperatively?

The general consensus, comprising reviews and one retired practice parameter by the American Academy of Neurology (AAN), is that AEDs should not be routinely used for prophylaxis [3, 4, 7, 8, 30], and specific guidelines for the administration of AEDs in meningiomas are yet to be formulated. As a result, a wide variety of AED practices are observed, firstly at a local level in our institute and secondly on a wider scale as the AANS/CNS survey demonstrated [31]. AEDs in our study were only administered to 8.1% of seizure-naïve patients compared to 63% of surgeons prescribing AEDs almost always [31]. This highlights, that for the time being, AEDs will continue to be prescribed in the neurosurgical community, despite the lack of proven benefit.

Previous studies have devised scoring systems to guide AED prescribing including the STAMPE2 prognostic index [17]. The limitation of such scoring systems is that it’s difficult to estimate the reduction rate of seizures at each level and hence, the choice of a cut-off point for treatment is arbitrary. Our solution to this was to model data using survival analysis, to estimate the effect of AEDs in patients with different combinations of independent risk factors in all patients and in those seizure-naïve specifically. The hazard ratios for AED treatment in the models equate to an approximate seizure reduction rate of 40%. Although this was not statistically significant, these data could help direct the administration of AEDs, which due to side effects and impact on QoL, should not be prescribed routinely.

### Choice of AED and duration of treatment

The wide variation in AED choice and duration of use in our study limits analysis of which drug might be most effective. Studies addressing optimal AED regimens are required, specifically for preoperative epilepsy patients whose seizures cease to recur for the first 2 weeks after surgery. In our study, this was observed in 49 patients of which 32 (65.3%) were on AEDs for up to 12 months. Recommendations are to allow a duration of at least 2 years of seizure-freedom before discontinuation is attempted [32]; however, this is based on AED trials that almost invariably exclude brain tumour patients from their populations, and therefore this cannot be applied to meningioma patients. The question of how long to continue AEDs could pragmatically be based around driving regulations, adverse events and QoL. Targeting a policy of 3 or 12 months of AED administration would be achieved in the context of a RCT. We could not draw any meaningful conclusions to support the use of one drug prophylactically over others. A well-designed trial is also required to address this question.

### Postoperative seizure freedom

Through 12 months of follow-up, the probabilities of seizure-freedom in seizure-naïve patients and preoperative epilepsy patients were approximately 90 and 80% respectively. Beyond 12 months, the rate in patients with preoperative epilepsy dropped to 72.1%. Within 12 months of seizure-onset, likelihood of seizure freedom was 44.4% among subjects with preoperative seizures and 72.4% in patients without them. This implies that whilst an acceptable rate of seizure-control could be achieved in seizure-naïve patients, control of seizures in patients with preoperative epilepsy is more challenging. The ILAE’s definition of drug resistant epilepsy emphasises that treatment failure is assessed in the context of two trialled drugs, either in combination or as monotherapies [33]. In our study, 61.1% of patients with uncontrolled seizures did not meet the aforementioned criteria. Those rates therefore need to be further evaluated following escalation of AED treatment.

### Study limitations

This is a retrospective study of uneven groups operated for a meningioma in a single institution. AED choice and duration varied across patients and drug-related side effects were not recorded, therefore comparisons of drugs could not be performed. Seizure types are likely to impact patients differently however seizure semiology postoperatively was not recorded. Lastly, parameters of the three BLR models were acceptable for two and poor for the model pertaining to seizure-naïve patients.

## Conclusions

**Fig. 2:**
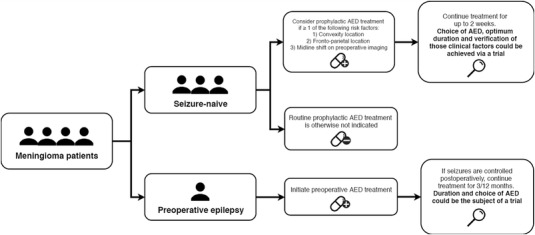
Recommendations for treatment and future research

Summarised in Fig. 2 are our recommendations for treatment and future research. Seizures and AEDs in meningioma patients have a great impact on QoL. The ability to identify patients at risk of seizures and to understand how AEDs augment their risk is of importance to clinicians and patients. Convexity and fronto-parietal locations as well as preoperative epilepsy are the factors most strongly related to postoperative seizures, in addition to the presence of a midline shift on preoperative imaging in seizure-naïve patients. AEDs could potentially prove beneficial in those groups of patients with an approximate seizure-reduction rate of 40%. High quality randomised controlled trials however are required to verify these factors and to determine whether AEDs have a definitive role in reducing seizure rates postoperatively.
